# Overlapping between squamous cell carcinoma and mucocutaneous leishmaniasis in a diabetic patient

**DOI:** 10.1093/omcr/omad153

**Published:** 2024-02-16

**Authors:** Hala Khaddam, Kheder Kheder, Rami Sabouni, Younes Al Mahmoud, Rasha Nabhan

**Affiliations:** Faculty of Medicine, Tartous University, Tartous, Syria; Department of Ophthalmology, Al Basel Hospital, Tartous, Syria; Faculty of Medicine, Damascus University, Damascus, Syria; Neurology Department, Tishreen University Hospital, Latakia, Syria; Department of Dermatology, Duraykish National Hospital, Tartous, Syria; Board Certification in Skin Diseases, Damascus University, Damascus, Syria

**Keywords:** Infectious diseases and tropical medicine, Dermatology

## Abstract

Mucocutaneous leishmaniasis (MCL) is a leishmania infection; that usually affects the oral and nasal mucosa. The coexistence of leishmania and malignancy is rarely reported and mainly in immunocompromised patients. We report a case of an overlapping between leishmania and squamous cell carcinoma (SCC) in a 60-year-old immunocompetent Syrian female. The patient presented with a one-year crusty nodule on the lower lip. Since she lives in an endemic region, leishmaniasis was suggested, and confirmed with a Giemsa-stained smear. After 20 days of meglumine antimoniate treatment, the patient revealed no signs of recovery, thus, the treatment was prolonged with the addition of sodium stibogluconate injections. Later, she presented with increased symptoms. A biopsy was performed due to SCC suspicion, and it was verified. The patient underwent complete surgical removal of the lesion. After a one-year follow-up, no recurrence was observed. We illustrated the importance of considering SCC in cases of refractory leishmaniasis.

## INTRODUCTION

Leishmaniasis is a parasitic disease with an incidence rate of about one million cases and a tendency to spread in low-income countries; among poor environs in particular [1]. Leishmaniasis is classified depending on the locus of the lesion into three clinical manifestations [2]. Mucocutaneous leishmaniasis (MCL) is typically found in the upper respiratory tract, involving the oral and nasal mucosa, with occasional involvement of laryngeal and pharyngeal mucosa [3]. Its destructive tendency to these sites, ranked MCL as a life-threatening condition [1].

MCL is an extension of Leishmania braziliensis parasite from cutaneous into mucosal sites, usually following substandard treatment of Cutaneous Leishmaniasis with a latent period of months, even years [2]. Some MCL cases were reported as primary lesions affecting mostly immunocompromised patients. The diversity of MCL clinical manifestations, including erythema, papules and exophytic nodules, corresponds with a wide spectrum of differential diagnoses, involving fungal diseases tuberculosis, leprosy and squamous cell carcinoma (SCC) [3].

Due to the rare presentation of MCL in the perioral area; confirmatory diagnosis of lip leishmaniasis can be challenging [2]. This diagnostic challenge can present as a misdiagnosed SCC [4, 5]. Although the superimposition of SCC on a previous leishmania lesion was previously reported, the coexistence of malignancy and leishmania lesion is less discussed [6]. Such rare presentation was reported in cases of immunocompromised patients, attributing this phenomenon to the dysfunctional immunity [7]. Thus, this case illustrates a primary MCL of the lower lip that overlapped with SCC in a diabetic patient.

## CASE PRESENTATION

A 60-year-old female with a history of vitiligo, well-controlled diabetes and hypertension, presented to the dermatology clinic with a painful, itchy mass of one-year duration, affecting the right lateral part of the lower lip. The lesion appeared one year ago as a small nodule, with a history of an insect bite one-month before the appearance of the lesion. The lesion was painful and itchy. The physical examination showed a 4 cm, solitary, tender, rounded, crusty nodule with spontaneous bleeding upon palpation. The lower lip was edematous and erythematous. No other skin lesions were detected.

**Figure 1:**
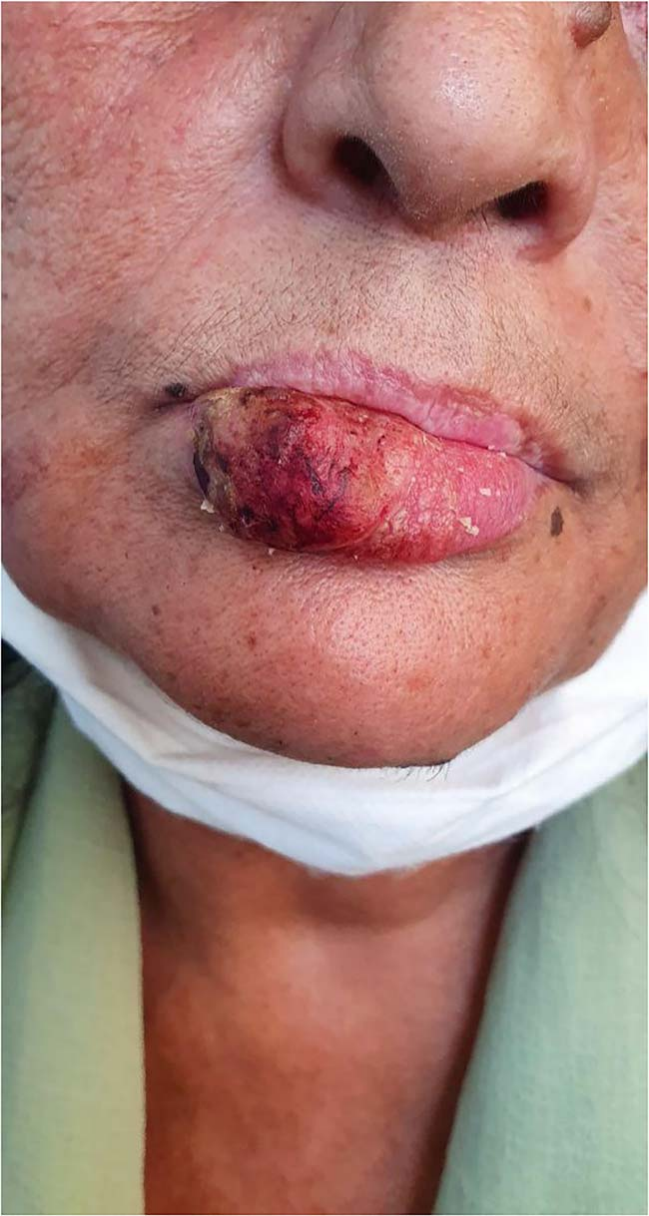
Crusted and partially ulcerated swelling of the right lower lip.

The clinical presentation suggested a differential diagnosis of SCC and leishmaniasis. According to the epidemiological context, we referred the patient to the department of leishmania and other contagious diseases due to high suspicion of leishmania infection. A Giemsa-stained smear was carried out and was positive for leishmania. Accordingly, twenty, daily, intramuscular injections of meglumine antimoniate were started. Upon follow-up, we noticed no signs of improvement. Thus, the injections were prescribed for another 15 days, followed by application of intramuscular injections of sodium stibogluconate daily for 20 days. However, in the last 10 days of the recommended protocol, the patient did not adhere as she complained of severe pain. Subsequently, a new assessment was carried out in the dermal clinic and revealed a 3 × 2 cm soft, crusted and partially ulcerated swelling on the lower lip (Fig. 1).

**Figure 2:**
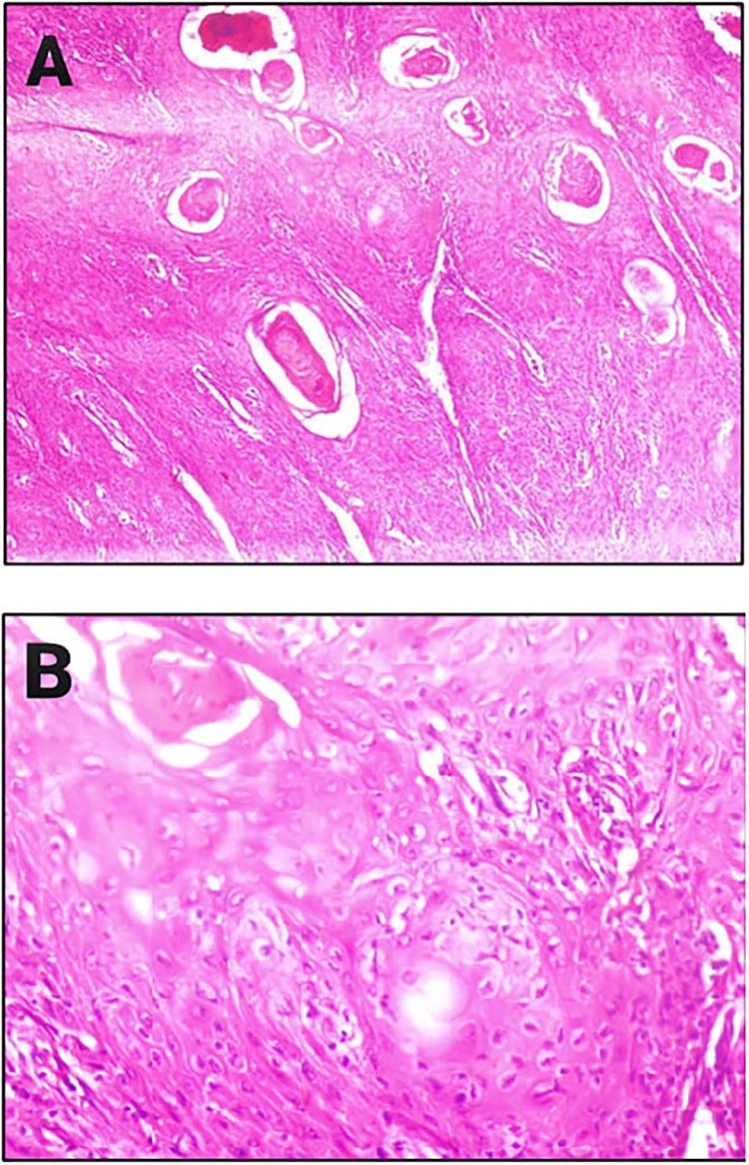
(**A**) Histopathological examination of the lesion showing keratin pearls (hematoxylin and eosin, original magnification ×20). (**B**) Histopathological examination of the lesion showing intracellular bridges and infiltrating atypical cells consisting of polygonal cells with mitotic activity and amounts of eosinophilic cytoplasm, nuclear enlargement, hyperchromasia (hematoxylin and eosin, original magnification ×40).

Liquid nitrogen cryotherapy alongside 3-day allopurinol (300 mg/day, 600 mg/day and 900 mg/day) were added to the previous protocol. Nevertheless, the patient was lost to follow-up due to low access to hospitals in her region. She returned after one month with an enlarged lesion measuring 5 cm and increased pain. Accordingly, a biopsy was performed due to SCC suspicion. The biopsy showed a well-differentiated SCC grade I. (Fig. 2). Computerized tomography scan showed no signs of lymphadenopathy.

**Figure 3:**
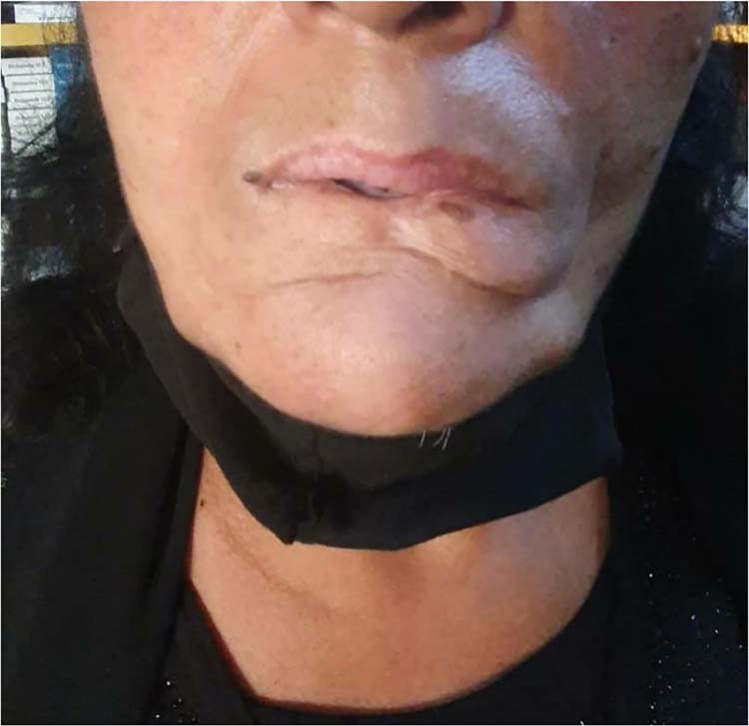
Post-surgical, one-year follow-up.

The patient underwent a complete surgical removal of the lesion and lip reconstruction. Histopathological examination verified the diagnosis of grade II SCC and revealed absent tumor infiltration in peripheral and deep resection margins. Thus, the final diagnosis was SCC grade II Stage (III). A one-year follow-up revealed no recurrence and sustained normal lip function based on clinical examination. (Fig. 3).

## DISCUSSION

Perioral MCL can present as a nodular swelling, long-standing ulcers, or slowly growing nodules [3, 4]. It can also present with cervical lymphadenopathy [3]. This neoplastic-like presentation can overlap with malignancies, especially that perioral MCL is relatively rare, making it less suspected. Thus, misdiagnosis of leishmaniasis as SCC was previously reported [4].

The reported period between leishmania infection and discovering a malignancy is approximately between two to five years [7]. However, in our case, the diagnosis of SCC was made two months after the initial diagnosis of MCL, which would raise suspicion of the diagnosis of leishmania. However, the reported history of being bitten by an insect, living in an endemic area, and positive leishmania test excludes a misdiagnosed MCL. Rather, the short period of the diagnosed SCC after the leishmania infection, supports the probability of having a coexistence of malignancy and MCL.

Leishmania can promote atypical squamous cell proliferation [8, 9], which might further complicate the diagnostic challenge. Such presentation was previously reported in one case, in which the cutaneous leishmaniasis presented as abnormal squamous proliferation indicating a diagnosis of well-differentiated SCC. The lesion responded to leishmania treatments with total recovery, and no signs of epithelial proliferation on the follow-up biopsy [9]. The failure to respond to leishmania treatment raises suspicion toward either resistant leishmania or malignancy [6]. Although Our patient received multiple leishmania treatments, the lesions were refractory to treatments, which supports the co-exitance of leishmania and malignancy.

The superimposition of malignancy on leishmania lesions was previously reported [6]. However, the association between malignancy and leishmania is yet to be known [7]. A systematic review found no association between malignancy and leishmania. However, the literature lacks properly designed studies since this systematic review was based on case reports [10]. Such coexistence of leishmaniasis and malignancy is suggested to be caused by the impaired immunity in immunocompromised patients [6, 7]. Nevertheless, our patient showed no signs of immunocompromisation.

MCL can present with a wide variety of manifestations, especially when presenting in the perioral area, which can lead to a challenging diagnosis and misdiagnosis. However, the positive history alongside the response to leishmania drugs would erase such suspicion, with resistance to drugs being in mind. Nevertheless, if the diagnosis is still vague and the biopsy corresponds with the findings of SCC, it would be better to be managed as a tumor to eradicate the possibility of tumor spreading as much as possible.
